# IterTunnel; a method for predicting and evaluating ligand EgressTunnels in proteins with buried active sites

**DOI:** 10.1186/1758-2946-6-S1-P62

**Published:** 2014-03-11

**Authors:** Laura J Kingsley, Markus A Lill

**Affiliations:** 1 Department of Medicinal Chemistry and Molecular Pharmacology, Purdue University, West Lafayette, IN, 47906, USA

## 

The computational prediction of ligand entry and egress paths in proteins has become an emerging topic in computational biology due to the potential for estimating kinetic properties of drug binding. These properties are related to important pharmacological quantities such as the k_on_ and k_off_ rate of drugs [1,2].We have investigated the influence of protein flexibility on tunnel prediction using geometric methods by comparing tunnels identified in static structures with those found in structural ensembles of three CYP isozymes. We found drastic differences between tunnels predicted in the crystal structures as opposed to those predicted in the ensembles [3]. Furthermore, we found significant differences between tunnels identified in the apo versus the holo protein ensembles [3].

While geometric prediction provides a good starting point for tunnel prediction, in order to estimate kinetic properties, more detailed investigations of the ligand binding process are required. We have developed a tunnel prediction methodology, IterTunnel, which predicts tunnels in proteins and estimates the free energy of ligand unbinding using a combination of geometric tunnel prediction with steered molecular dynamics and umbrella sampling [4]. Applying this new method to cytochrome P450 2B6 (CYP2B6), we demonstrate that the ligand itself plays an important role in reshaping tunnels as it traverses through a protein. This process results in the exposure of new tunnels and the closure of pre-existing tunnels as the ligand migrates from the active site. We found that many of the tunnels that are exposed due to ligand-induced conformational changes are amongst the most energetically favorable tunnels for ligand egress in CYP2B6 [4].
